# Comparison of reactive oxygen species in neat and washed semen of infertile men

**Published:** 2014-05

**Authors:** Mohammad Reza Moein, Serajedin Vahidi, Jalal Ghasemzadeh, Nasim Tabibnejad

**Affiliations:** *Research and Clinical Center for Infertility, Shahid Sadoughi University of Medical Sciences, Yazd, Iran.*

**Keywords:** *ROS*, *Neat sperm*, *Washed sperm*, *Pyospermia*

## Abstract

**Background:** Male are involved in near 50% of cases of infertility and reactive oxygen species (ROS) playing an important role in decreasing fertility potential. Accurate measurement of ROS seems to be important in evaluation of infertile male patients.

**Objective:** To compare ROS measurement in neat and washed semen samples of infertile men and define the best method for evaluation of ROS in these patients.

**Materials and Methods: **We measured the level of ROS in semen samples of thirty five non-azoospermic men with infertility. The semen samples were divided into two parts and the semen parameters and ROS levels in neat and washed samples were evaluated. We also evaluated the presence of pyospermia using peroxidase test.

**Results: **The differences regarding sperm count and quick motility were significant in neat and washed semen samples. The mean ROS level was significantly higher in neat samples compared with washed spermatozoa (7.50 RLU vs. 1.20 RLU respectively). Difference in ROS levels was more significant in patients with pyospermia compared to whom with no pyospermia (378.67 RLU vs. 9.48 RLU respectively).

**Conclusion:** Our study confirmed that neat or unprocessed samples are better index of normal oxidative status of semen samples. Because we do not artificially add or remove factors that may play an important role in oxidative equilibrium status.

## Introduction

Infertility affects about 15% of couple during their reproductive years and male factors are involved in about half of the cases (1). In approximately 25% of males, the cause of infertility cannot be determined (2). In these cases standard seminal analysis does not detect any abnormalities. But, the routine measurements do not show seminal defects at molecular levels that might be induced by reactive oxygen species (ROS), which are associated with male infertility (3). 

During the past few decades the role of high levels of ROS as a major cause of male infertility has got more attention (4). ROS generated by spermatozoa play an important role in normal physiologic processes like acrosome reaction, sperm capacitation, oocyte fusion and mitochondrial stability (5-9). But, abnormal production of ROS by spermatozoa and leukocytes could cause oxidative stress, which is harmful to spermatozoa, and negatively affects the fertilizing potential of spermatozoa and normal pregnancy rate (10). It has also shown that high level of ROS significantly lower success rate of in vitro fertilization (IVF) (11). 

Between 25-88% of infertile men have elevated ROS levels in their semen (12). So the importance of developing good and reliable tests to measure level of ROS is obviously clear. There are different methods for measurement of ROS In seminal plasma. ROS can be measured both in neat and washed semen samples (13, 14). Some studies showed that semen processing including vortexing and centrifugation could increases the level of ROS in seminal fluid (15). They postulate that when seminal plasma is extracted during washing procedures, so too are protective antioxidant. As a result, level of ROS increase in the sample (16). But, on the other hand, extracting some of major contributors to ROS production in seminal fluid after washing could cause decreasing level of ROS in these samples. So, in this study we examined ROS levels in neat and washed semen samples in subfertile men referring to our center to see: 1) is there any difference between ROS level in neat and washed semen samples 2) what are the probable sources of this difference. 

At last we will conclude which of these methods could be a better predictor of infertility for natural conception, and also for couples who are candidate of assisted reproductive techniques (ART).

## Materials and methods

**Patients**

This experimental study has been carried out in Yazd Research and Clinical Center for Infertility Thirty five semen samples were obtained from 35 men who were candidate for enrollment in infertility treatment cycles. Azoospermic patients and men who had severe oligozoospermia and history of taking antioxidant medication within the last three months were excluded from the study. Semen parameters and ROS level were evaluated in neat and washed semen samples. Peroxidase (ENDtZ) test was done on all semen samples and samples with one million WBC/ml or more were considered positive for pyospermia. A written informed consent was obtained from the patients. This experimental prospective study has been approved by the Ethics Committee of Research and Clinical Center for Infertility, Shahid Sadoughi University of Medical Sciences.

**Semen analysis**

All specimens were collected by masturbation at the andrology laboratory, after an abstinence period of 48-72 hours. After complete liquefaction in room temperature, routine semen analysis was performed manually according to 5^th ^edition of WHO guidelines. For each measurement, a 5 µL aliquot from patients sample was loaded into a microcell chamber (Conception technologies, San Diego, CA) and analyzed for sperm concentration and motility. Seminal smears were stained with Diff-Quick stain and sperm morphology was assessed according to WHO criteria (17).

**ROS** **measurements by chemiluminescence** **assay**

An aliquot of liquefied neat semen was used for the primary measurement of ROS. For evaluation of ROS in washed specimens, semen specimens were centrifuged at 300×g for 7 min and seminal plasma was removed. The pellet was washed with phosphate buffered saline (PBS) and re-suspended in the same media. 10 ml of luminol (5-amino-2, 3 dihydro-1, 4 phtalazindione; Sigma chemical Co., USA) used as a probe and was added to the aliquot. A negative control was prepared by adding 10 micro liter of PBS. The ROS levels were assessed by measuring chemiluminiscence activity with an Autolamat LB 935 Luminometer (Berthold technologies, Bad-wildbad, Germany) in the integrated mode for 15 minutes. The results were expressed as RLU (Relative light unit) per 20 million spermatozoa (13).

**White blood cell (ENDtZ) test**

The leukocyte concentration in the specimen was detected by the ENDtZ (peroxidase) test (18). First, semen samples were mixed well. Then 20µL of fresh, liquefied semen mixed with the same volume of PBS and 40 µL of benzidine solution )4,4′-Diaminobiphenyl; Sigma chemical Co., USA) was added later to the mixture. 

The mixture was vortexed and put into a foiled covered tube and allowed to sit in room temperature for 5 min. Five µL of this mixture was placed in a makler chamber and examined for dark brown stained cells. Brown stained cells indicating the presence of peroxidase were considered leukocyte. Samples with more than 1×10^6^ WBC s/ml of semen were considered positive. Patients were divided in two groups according to presence or absence of pyospermia. ROS levels were checked in each group.

**Statistical analysis**

The Statistical Package for the Social Sciences 15.0 software was used to analyze the data of all patients. Data were expressed in mean±SD. For comparisons between neat and washed semen samples, Wilcoxon Signed Ranks test was used. P<0.05 was considered statistically significant.

## Results

The mean age of subjects was 31.43±6.60 years and the duration of infertility was 5.20±4.17 years. Sperm parameters including sperm count, quick and slow progressive motility before and after sperm processing are shown in table I. The differences regarding sperm count and quick motility were significant. The mean ROS level was significantly higher in neat samples compared with washed spermatozoa (7.50 RLU vs. 1.2 RLU) (p=0.010) (Table II). ROS levels regarding presence or absence of pyospermia are presented in table III.

**Table I:** Comparison of sperm parameters and ROS levels between neat and washed spermatozoa

**Variable**	**Neat sperm**		**Washed sperm **		**p-value** *
	**Mean**	**S.D.**	**Mean**	**S.D.**	
Sperm count (mil/ml)	95.94	57.50	57.57	31.62	0.003
Quick motility (%)	17.82	18.43	49.37	33.67	0.000
Slow motility (%)	31.14	8.56	28.91	17.49	0.494
Sperm morphology (%)	33.17	20.23			

N=35.

* Wilcoxon Signed Ranks test.

**Table II:** Comparison of ROS levels between neat and washed spermatozoa

**Group**	**ROS (RLU)**		**p-val**ue**
	**Neat sperm**	**Washed sperm**	
Total (n=35)	7.50	1.20	0.010
*Endtz Positive (n=6)	264.35	7.15	0.032
Endtz Negative ( n=29)	4.80	1.20	0.498

Data are presented as median

* Endtz AW, 1974.

** Wilcoxon Signed Ranks test

**Table III:** Comparison of ROS levels among men with pyospermia

**ROS (RLU)**	**Neat sperm (n=6)**	**Washed sperm (n=6)**	**p-value** *
Mean ± SD	378.67 ± 312.66	9.48±9.30	0.032
Q1	116.00	0.55	
Median	264.35	7.15	
Q3	688.53	20.75	

Q1: First quartile (splits off the lowest 25% of data from the highest 75%)

Q3: Third quartile (splits off the highest 25% of data from the lowest 75%)

* Wilcoxon Signed Ranks test

## Discussion

About fifty percent of infertility problems are attributed to male factors. In spite of major advances in diagnosis and treatment of male infertility, no specific etiology can be found in many of these men (2). One of the etiologies, which in recent years paid more attention to, is elevated level of ROS in seminal fluid. These free radicals produced by spermatozoa and leukocytes and are scavenged by various antioxidants in seminal plasma. It seems that many infertile patients with diagnosis of unexplained or idiopathic infertility may suffer from oxidative stress, as previous studies showed elevated levels of these radicals in 25-70% of these patients (20). 

Oxidative stress can affect many sperm functions like: impaired motility, increased apoptosis and sperm chromatin damage (21, 22). It also increases the rate of pregnancy loss. Different mechanisms have been proposed for the elevation of ROS and producing oxidative stress. Leukocytes and immature germ cells are two major source of ROS production in seminal fluid. Elevated levels of these cells in semen could induce oxidative stress (21). There are some well-known conditions that implicated as a cause of ROS elevation and inducing oxidative stress like: Infection, inflammation, smoking, varicocele and spinal cord injury (23-25).

Due to importance of ROS and oxidative stress in evaluating infertile men, it seems necessary that infertility centers and laboratories involved in this field will develop and standardize the methods of ROS measurements and also determine the normal values for these parameters. Nowadays, the most direct assay for evaluating ROS is by Luminometer, which works on the basis of photo emission from particular cells (13). At first, most of times these tests were done on processed or washed sperm. But, later some studies showed that seminal manipulations during sperm processing, and also sperm preparation techniques can induce elevation of free radicals in seminal fluid (15). 

So, later studies assessed the ROS levels in neat sperm samples. They showed that determining ROS level in neat samples is more prognostic of men natural status and so they advised measuring ROS level in neat sperm. Also increased levels of ROS in processed samples, used for ART, implicated as a cause of failure of reproductive techniques (26). Generally ROS levels can be measured in neat semen, washed sperm solution and after sperm preparation techniques (16, 27, 28).

In this study we worked on thirty five semen samples from infertile men referring to our infertility center. We divided the samples in two parts. First part of samples were wholly unprocessed and ROS level was measured in these neat semen. Sperm processing and washing done for the second part of samples and again ROS levels were checked in these processed samples. Also as mentioned, we did peroxidase test on all samples. Our study showed that ROS levels in processed samples were significantly lower than neat semen that is in opposite to some previous studies. 

These studies stated that multiple centrifugation, resuspension and vortexing steps involved in these procedures artificially elevate ROS levels (15). Also they reasoned that removing seminal plasma, which contains high amounts of antioxidants, during semen processing, is another reason for elevation of ROS levels in these samples. But, on the other hand as leukocytes are a major source of ROS production in semen and washing and processing of semen cause decreased levels of leukocyte in semen samples, this may be explained decreased level of ROS in processed samples. As it shown on table II this effect is more significant in samples with abnormal level of leukocyte. One limitations of our study was that we didn't count and consider the leukocyte number below the normal accepted value which is less than 10^6^/ml.

## Conclusion

In conclusion our study like previous ones confirmed that the best samples for evaluation of normal oxidative status of semen samples are neat or unprocessed samples. Because we don't artificially add or remove factors that play an important role in oxidative equilibrium status. Of course is expectable that we may have abnormal elevation of free radicals in some samples that be used for ART, and so must try to remove or decrease their levels to increase ART success rate.
